# lncRNA MALAT1-mediated regulation of cholesterol-oxidative stress-iron metabolic dysregulation by paeoniflorin in osteoarthritic chondrocytes

**DOI:** 10.1080/13880209.2026.2620862

**Published:** 2026-02-12

**Authors:** Changlong Fu, Shujie Lan, Yanming Lin, Danling Chen, Zhenzhen Yang, Changxian Chen, Yue Chen, Yijing Yuan, Weihong Zhong, Qing Lin, Zhiqiang Huang

**Affiliations:** a Academy of Integrative Medicine, Fujian University of Traditional Chinese Medicine, Fuzhou, Fujian, China; b Fujian Provincial Key Laboratory of Integrative Medicine on Geriatrics, Fuzhou, Fujian, China; c First School of Clinical Medicine, Fujian University of Traditional Chinese Medicine, Fuzhou, Fujian, China; d Department of Pharmacy, Quanzhou Orthopedic-traumatological Hospital, Quanzhou, Fujian, China; e School of Orthopedics and Traumatology, Fujian University of Traditional Chinese Medicine, Fuzhou, China; f Quanzhou Hospital of Traditional Chinese Medicine, Quanzhou, Fujian, China

**Keywords:** Paeoniflorin, lncRNA MALAT1, osteoarthritis, metabolism

## Abstract

**Context:**

Osteoarthritis is a chronic degenerative disease characterized by metabolic dysregulation, inflammation, and oxidative stress. MALAT1 plays a key role in OA pathogenesis. Pae exhibits anti-inflammatory activity, but its regulatory role in cholesterol-oxidative stress-iron metabolism in chondrocytes remains unclear.

**Objective:**

To investigate whether Pae can alleviate cholesterol-oxidative stress-iron metabolic dysregulation in OA chondrocytes *via* modulation of MALAT1.

**Materials & methods:**

OA was induced in mice using a modified Hulth method, followed by intra-articular injection of MALAT1 overexpression plasmid. Cartilage morphology and the expression of MALAT1 and related genes/proteins were assessed by histology, RT-PCR, and Western blot. *In vitro*, IL-1β-treated chondrocytes were used to model OA, and MALAT1 knockdown was achieved *via* lentiviral transfection. FISH, RT-PCR, Western blot, and flow cytometry were used to evaluate the effects of Pae on molecular markers and apoptosis.

**Results:**

**In vivo**

In vivo, Pae improved cartilage morphology and restored the expression of key genes and proteins, whereas MALAT1 overexpression attenuated these effects. In IL-1β-treated OA chondrocytes, Pae reversed abnormal mRNA expression of MALAT1 and related genes. FISH analysis showed that Pae reduced MALAT1 fluorescence in chondrocytes. Western blot revealed that Pae significantly restored protein expression in sh-MALAT1 chondrocytes. Furthermore, flow cytometry analysis revealed that Pae intervention significantly reduced the apoptosis rate of IL-1β-induced chondrocytes from 34.57% to 27.74%. sh-MALAT1 resulted in a more pronounced reduction in chondrocyte apoptosis, with the apoptotic rate further decreasing to 13.21%.

**Discussion and Conclusion:**

Paeoniflorin ameliorates cholesterol-oxidative stress-iron metabolic dysregulation in OA chondrocytes *via* modulation of MALAT1, providing mechanistic insight into its potential therapeutic effects.

## Introduction

The most common form of arthritis, osteoarthritis (OA), is characterized by telltale changes in joint tissues, such as swelling of the joints, inflammation of the synovial membrane, and the slow degeneration of articular cartilage (Kraus et al., 2015; Cheng et al., 2019; Nogueira-Recalde et al., 2025).OA severely affects patients’ physical function and mental health, with about 7% of the global population suffering from the disease, making it a leading cause of adult disability (Rat et al., 2025; Srivastava, 2023; Safiri et al., 2020). Current treatments—such as analgesics, nonsteroidal anti-inflammatory drugs, and joint replacement—offer limited benefits for cartilage repair and symptom relief, highlighting the urgent need for more effective therapies. Pae, a bioactive compound from the traditional Chinese herb Paeonia lactiflora, is the principal pharmacological component underlying its therapeutic effects. Pae exerts potent immunomodulatory, anti-inflammatory, and antioxidant activities (Chen et al., 2021; Zhang et al., 2020; Wu et al., 2007; Jiao et al., 2021). In chondrocytes, Pae suppresses reactive oxygen species (ROS) production, decreases extracellular matrix degradation and chondrolytic enzyme secretion, reduces apoptosis, and thereby provides a strong chondroprotective effect (Liao et al., 2018; Cheng et al., 2025; Yao et al., 2020).

In recent years, accumulating evidence has demonstrated that lncRNA MALAT1 participates in key pathological processes of OA, including inflammation and apoptosis, and is markedly upregulated in obese OA patients, indicating a potential link between lncRNA MALAT1 and metabolic dysregulation (Nanus et al., 2020). Obesity is recognized as a major risk factor for OA, and obesity-associated lipid metabolic abnormalities can further accelerate OA progression (Sobieh et al., 2023). Importantly, maintaining lipid metabolic homeostasis has been shown to suppress extracellular matrix degradation in chondrocytes, reduce the production of inflammatory mediators, and alleviate subchondral bone degeneration (Mei et al., 2025; Li et al., 2025).

Increasing attention has been given to the role of microenvironmental homeostasis in OA progression, with oxidative stress emerging as a key therapeutic target. Studies have shown that alleviating oxidative damage can significantly promote cartilage repair and regeneration (Zhang et al., 2019; Zou et al., 2025). Ferroptosis, a distinct non-apoptotic form of regulated cell death, is closely associated with lipid metabolism and oxidative stress (Dixon, 2024). Disruption of iron homeostasis leads to iron overload and excessive lipid ROS accumulation in chondrocytes, which further impairs antioxidant defense systems, damages the microarchitecture of subchondral bone, and ultimately induces ferroptotic cell death, thereby promoting OA progression.

Recent evidence indicates that cholesterol metabolic dysregulation can directly trigger intracellular oxidative stress (Wu et al., 2025), and when oxidative stress becomes excessive, it can further drive ferroptosis (Zhang et al., 2022). These findings suggest that cholesterol metabolism disorders, oxidative stress, and ferroptosis are not isolated pathological events, but may form a continuous metabolic regulatory axis that collectively contributes to chondrocyte dysfunction and OA progression.

Moreover, maintaining intracellular metabolic homeostasis has been shown to be essential for OA prevention (Zhang et al., 2019). Based on our previous findings, we have preliminarily revealed a link between cholesterol-iron metabolism and OA. However, the molecular mechanisms underlying the cholesterol-oxidative stress-iron metabolic axis in OA remain to be fully elucidated. In osteoarthritic chondrocytes, a continuous dysregulation of the cholesterol-oxidative stress-iron metabolic axis may exist, in which lncRNA MALAT1 may play an important role, mediating this process to promote cartilage degeneration. Whether paeoniflorin (Pae) can serve as a potential therapeutic strategy to ameliorate this metabolic imbalance in OA also warrants further investigation.

## Materials and methods

### Primary reagents

Paeoniflorin (purity ≥98%, C_23_H_28_O_11_, Lot#: C16982607, CAS: 23180-57-6) was purchased from Shanghai Macklin Biochemical Co., Ltd. The supplier verified the identity and purity of the compound using rigorous analytical methods, including high-performance liquid chromatography (HPLC) and nuclear magnetic resonance (NMR), to ensure its authenticity and quality for experimental use; Isoflurane (R510-22; RWD Life Technology Co., Ltd., China); Hematoxylin-Eosin, Fuchsin-Golgi Stain Kit, Toluidine Blue Stain Solution, and Masson’s Trichrome Stain Solution from Beijing Solarbio Biotechnology Co., Ltd., China; SuperLumia ECL Kit (Meirun Bio Co., Ltd., China); and Fetal Bovine Serum (Thermo Fisher Scientific Inc., USA); Isoflurane (Shenzhen RWD Life Science Co., Ltd., Batch No. R510-22). We used antibodies against ABCA1, SREBP, CYP7B, CHOP, iNOS, COX2, ACSL4, and GPX4. All of these were from Proteintech (China, 00139339、00143435、00136020、00041108、00185131、00150356、00139404、00141308). A scrambled shRNA plasmid (pCMV-MCS-WPRE-Neo plasmid, Fuzhou Zaiji Biotechnology Co., Ltd.) was used as a negative control, and the detailed plasmid map is shown in Supplementary Figure S1.

### Animals

We used fifty SPF-grade male C57BL/6 mice, 8 weeks old and weighing between 20 and 25 g. The animals were provided by Shanghai Jihui Laboratory Animal Breeding Co., Ltd. [License No. SCXK (Hu) 2022-0009]. The animal study was conducted from July 11, 2025, to August 21, 2025.After a one-week acclimation period, we randomly assigned the animals to either the blank group (*n* = 10) or the experimental modeling group (*n* = 40). After induction of anesthesia with 5% isoflurane, the animals in the model group underwent local hair removal and disinfection around the knee joint. A longitudinal incision of approximately 2 cm was made along the medial margin of the patella and the medial femoral condyle. After exposing the knee joint, the anterior cruciate ligament and the medial collateral ligament were transected, and the medial meniscus was removed. The incision was then sutured and disinfected with povidone-iodine. The OA model was established using the modified Hulth surgical method (Li et al., 2020).

One week after surgery, we randomly divided the modeled animals into four groups: the model group (*n* = 10), the model + oe-MALAT1 group (*n* = 10), the model + Pae group (*n* = 10), and the model + oe-MALAT1 + Pae group (*n* = 10). Mice in the model + Pae group received oral Paeoniflorin (100 mg/kg/day) for 4 weeks (Wu et al., 2025). The model + oe-MALAT1 plasmid was administered intra-articularly to the experimental animals assigned to the oe-MALAT1 therapy; 20 μl was administered to each joint, for a total dose of 20 μg. On the other hand, the model and blank groups had a four-week oral gavage program using a comparable amount of saline solution. All of the individuals were anesthetized and mercifully decapitated at the end of the intervention session. After the intervention, the animals were euthanized under anesthesia by cervical dislocation. The knee articular cartilage was carefully isolated, placed into centrifuge tubes, and stored at −80 °C until further analysis. To minimize potential experimental bias, animals were randomly assigned to different experimental groups using a random number generator. All outcome assessments and data analyses were performed by investigators blinded to the group allocation. For *in vitro* experiments, treatments were applied in a randomized order, and all outcome analyses were performed without knowledge of the treatment groups. These measures ensured objective data collection and enhanced the reliability of the results. The number of mice per group was determined based on previous studies investigating cartilage repair in osteoarthritis models (Fu et al., 2025). Considering the design of our experiments and the requirement to perform each experiment in triplicate, the selected sample size was deemed sufficient to detect biologically meaningful differences while minimizing animal use. All experimental techniques were authorized by the Fujian University of Traditional Chinese Medicine Institutional Animal Care and Use Committee (Approval No. FJTCM IACUC 1N2025051).

### Morphological observation of cartilage tissue

After removal of excess muscle and connective tissues, knee joint specimens were fixed in 4% paraformaldehyde for 24 h. The tissues were then decalcified in ethylenediaminetetraacetic acid disodium salt (EDTA) solution for 7 consecutive days. Following graded ethanol dehydration, the samples were embedded in paraffin and sectioned. The paraffin sections were deparaffinized and subjected to multiple histological staining procedures, including hematoxylin and eosin (HE), Safranin O-Fast Green, toluidine blue, and Masson staining, according to the manufacturer’s instructions.

For HE staining, sections were first stained with hematoxylin, differentiated in differentiation solution, and then counterstained with eosin, followed by a quick rinse with distilled water. Safranin O-Fast Green staining was performed to assess pathological changes in mouse knee cartilage. Sections were initially stained with Weigert’s iron hematoxylin, differentiated in acidic differentiation solution, then stained with Fast Green and washed with weak acid solution, and finally immersed in Safranin O solution. For Masson staining, sections were stained with Weigert’s iron hematoxylin, differentiated in acidic differentiation solution, followed by staining with Masson blue, rinsed with distilled water, stained with acid fuchsin, treated with phosphomolybdic acid solution, and washed with weak acid solution. Toluidine blue staining was performed by immersing sections in a toluidine blue solution to visualize cartilage structures.

All stained sections were mounted with neutral resin. Tissue morphology was examined under a light microscope, and images were captured for further analysis. All procedures were performed strictly in accordance with the manufacturer’s instructions and standard protocols.

### Real-time PCR detection of mRNA levels in cartilage tissue

Cartilage tissues from each experimental group were placed in a mortar and ground in liquid nitrogen to obtain a homogeneous tissue powder. Total RNA was extracted using Trizol reagent according to the manufacturer’s instructions. Chloroform was added to remove impurities, mixed thoroughly, incubated, and centrifuged at 12,000 ×g. The supernatant was collected and mixed with an equal volume of isopropanol to precipitate RNA, followed by incubation and centrifugation. The RNA pellet was washed with 75% ethanol, centrifuged again, and the supernatant discarded. The resulting RNA was used for downstream analysis, and its concentration and purity were determined. Complementary DNA (cDNA) was synthesized from total RNA using the HiScript^®^ II Reverse Transcriptase kit. Reverse transcription was performed at 42 °C for 2 min, 50 °C for 15 min, and 85 °C for 5 min. The obtained cDNA was used for real-time quantitative PCR (qRT-PCR) to assess the expression levels of lncRNA MALAT1, ABCA1, iNOS, ACSL4, and CHOP in cartilage tissues. PCR amplification conditions were as follows: initial denaturation at 95 °C for 30 s, denaturation at 95 °C for 10 s, and annealing/extension at 60 °C for 30 s, for 40 cycles. Relative gene expression was calculated using the 2^−ΔΔCt^ method.The primer sequences are shown in Table 1.

**Table 1.** Primer sequence.

Gene	Primer(5′-3′)	Length/bp
*lncRNA MALAT*	Forward: 5′-GGCACTGAAGGCTTAATGTAGG-3′ Reverse: 5′-AAGGTGTTACGGTAGGGTAGTC-3′	135
*ABCA1*	Forward: 5′-GGTGGTGTTCTTCCTCATTACTG-3′ Reverse: 5′-TCACATCCTCATCCTCGTCATT-3′	107
*CHOP*	Forward: 5′-CTGGAAGCCTGGTATGAGGAT-3′ Reverse: 5′-CAGGGTCAAGAGTAGTGAAGGT-3′	121
*iNOS*	Forward: 5′-GGAGTGACGGCAAACATGACT-3′ Reverse: 5′-TCGATGCACAACTGGGTGAAC-3′	127
*ACSL4*	Forward: 5′-TGAACGTATCCCTGGACTAGG-3′ Reverse: 5′-TCAGACAGTGTAAGGGGTGAA-3′	141
*GAPDH*	Forward: 5′-ACGGCAAGTTCAACGGCACAG-3′ Reverse:5′-GAAGACGCCAGTAGACTCCACGAC-3′	189

### Western blot analysis in cartilage tissue

Cartilage tissues from each group were ground in liquid nitrogen and lysed using RIPA buffer supplemented with PMSF at a 100:1 ratio to extract total protein. Protein concentrations were determined using the BCA assay. Protein samples were mixed with loading buffer and denatured at 100 °C for 10 min in a metal bath.

Proteins were separated by SDS-PAGE and subsequently transferred onto PVDF membranes. Membranes were blocked at room temperature for 30 min using a rapid blocking solution to minimize nonspecific binding. PVDF membranes were then incubated overnight at 4 °C on a shaker with primary antibodies, including ABCA1 (1:1000), SREBP (1:1000), CYP7B1 (1:1000), CHOP (1:1000), iNOS (1:1000), COX2 (1:1000), ACSL4 (1:5000), GPX4 (1:1000), and β-actin (1:5000). After incubation with HRP-conjugated secondary antibody (1:5000) at room temperature, membranes were thoroughly washed with TBST. Protein signals were detected using a high-sensitivity ECL chemiluminescent substrate, and band intensities were quantified using densitometric analysis software. Relative expression levels of target proteins were normalized to β-actin by calculating the ratio of target protein intensity to β-actin intensity. All procedures were performed strictly following standard laboratory protocols to ensure accuracy and reproducibility.

### Total iron determination

The total iron content in bone tissues from each group was measured according to the manufacturer’s instructions of the total iron assay kit. Gradient iron standards and tissue homogenate samples were incubated with the chromogenic reagent at 37 °C for 40 min. The reaction mixtures were then centrifuged at 12,000 ×g for 10 min, and 300 μL of the supernatant was transferred to a 96-well plate. Absorbance was measured at 570 nm.

### Establishment of an in vitro chondrocyte OA model

We used twenty SPF-grade male C57BL/6 mice, aged 3 weeks. After euthanasia under anesthesia, we exercised the knee joint cartilage and cut it into ∼1 mm × 1 mm × 1 mm fragments. We washed fragments with PBS, digested them with trypsin, replaced the medium, and rinsed thoroughly. We then added 5 mL of 0.2% collagenase II solution and incubated the tissue at 37 °C in a 5% CO_2_ incubator to release chondrocytes. To establish an *in vitro* osteoarthritis model, we stimulated chondrocytes with IL-1β (10 μg/L) for 24 h (Wang et al., 2022). For intervention, cells in the model + Pae group were treated with Pae (50 μmol/L) for 48 h (Wu et al., 2025). All experimental techniques were authorized by the Fujian University of Traditional Chinese Medicine Institutional Animal Care and Use Committee (Approval No. FJTCM IACUC 1N2024113).

### Fluorescence in situ hybridization (FISH) detection in chondrocytes

We divided cells into four groups: blank, Pae, IL-1β, and IL-1β + Pae, according to the experimental design. We designed and synthesized a digoxin (DIG)-labeled lncRNA MALAT1 probe using the online platform (www.generalbiol.com). Each group’s chondrocytes were fixed in 4% paraformaldehyde and then incubated at 37 °C with 20 μg/mL proteinase K. Cells were then hybridized overnight with a mixed buffer containing the lncRNA MALAT1 probe. After hybridization, we performed gradient washes with sodium citrate solution to remove nonspecific binding. Subsequently, we blocked the samples and incubated them with the DIG-labeled probe. After washing, we applied CY3-TSA reagent and an anti-DIG-HRP antibody. We counterstained cell nuclei with DAPI (5 mg/L) and visualized the results using fluorescence microscopy to obtain images for analysis.

### Pae’s impact in IL-1β-induced chondrocytes under lncRNA MALAT1 knockdown

To investigate whether lncRNA MALAT1 regulates the OA cholesterol-oxidative stress-iron metabolic pathway, we silenced lncRNA MALAT1 expression by lentiviral transfection with the sh-MALAT1 plasmid. Based on our previous studies (Fu et al., 2024), a multiplicity of infection (MOI) of 12 provided the highest transfection efficiency while maintaining optimal cell viability. After transfection, we treated chondrocytes with Pae and assessed changes in the expression of lncRNA MALAT1, ABCA1, CHOP, iNOS, and ACSL4 using real-time PCR. At the end of each intervention, we removed cell supernatants, washed cells with PBS, and discarded the wash solution. We then lysed cells thoroughly with Trizol reagent. All subsequent steps, including RNA extraction, cDNA synthesis, and amplification, followed the procedures described in Section 2.4.

### Western blot analysis of pae effects on cartilage cell proteins in IL-1β-stimulated chondrocytes under lncRNA MALAT1 knockdown

We performed western blotting to evaluate changes in ABCA1, SREBP, CYP7B1, CHOP, iNOS, COX2, ACSL4, and GPX4 protein levels in sh-MALAT1-treated chondrocytes following Pae intervention. After each intervention, we lysed chondrocytes with RIPA buffer containing PMSF and scraped the lysates from culture dishes into EP tubes. We centrifuged the lysates and collected the supernatants, representing total cellular protein. Subsequent procedures, including quantification, electrophoresis, transfer, antibody incubation, and visualization, were conducted as described in Section 2.5.

### Flow cytometry analysis of pae’s effect on chondrocyte apoptosis rates

We assessed apoptosis in each group of chondrocytes using flow cytometry. Trypsin digestion was followed by centrifugation to extract the cells, two PBS washes, and resuspension in binding buffer. Samples were incubated for 20 min at room temperature in the dark after being stained with Annexin V-FITC and propidium iodide (PI) solutions. Next, we used flow cytometry to measure the rates of apoptosis.

### Statistical analysis

All experimental data were examined using SPSS 26.0 software. We used mean ± standard deviation (SD) to represent findings for data that fit a normal distribution. We used one-way analysis of variance (ANOVA) to compare groups.When variances were homogeneous, we performed least significant difference (LSD) post hoc tests; when variances were unequal, we applied the Games-Howell test. We considered differences statistically significant at *p* < 0.05. All experiments were performed with at least three independent biological replicates. Quantitative data are presented as bar graphs based on independent biological replicates, and the definition of error bars is specified in the corresponding figure legends.

## Results

### Pae exerts protective effects on articular cartilage in OA mice

**Figure 1.**
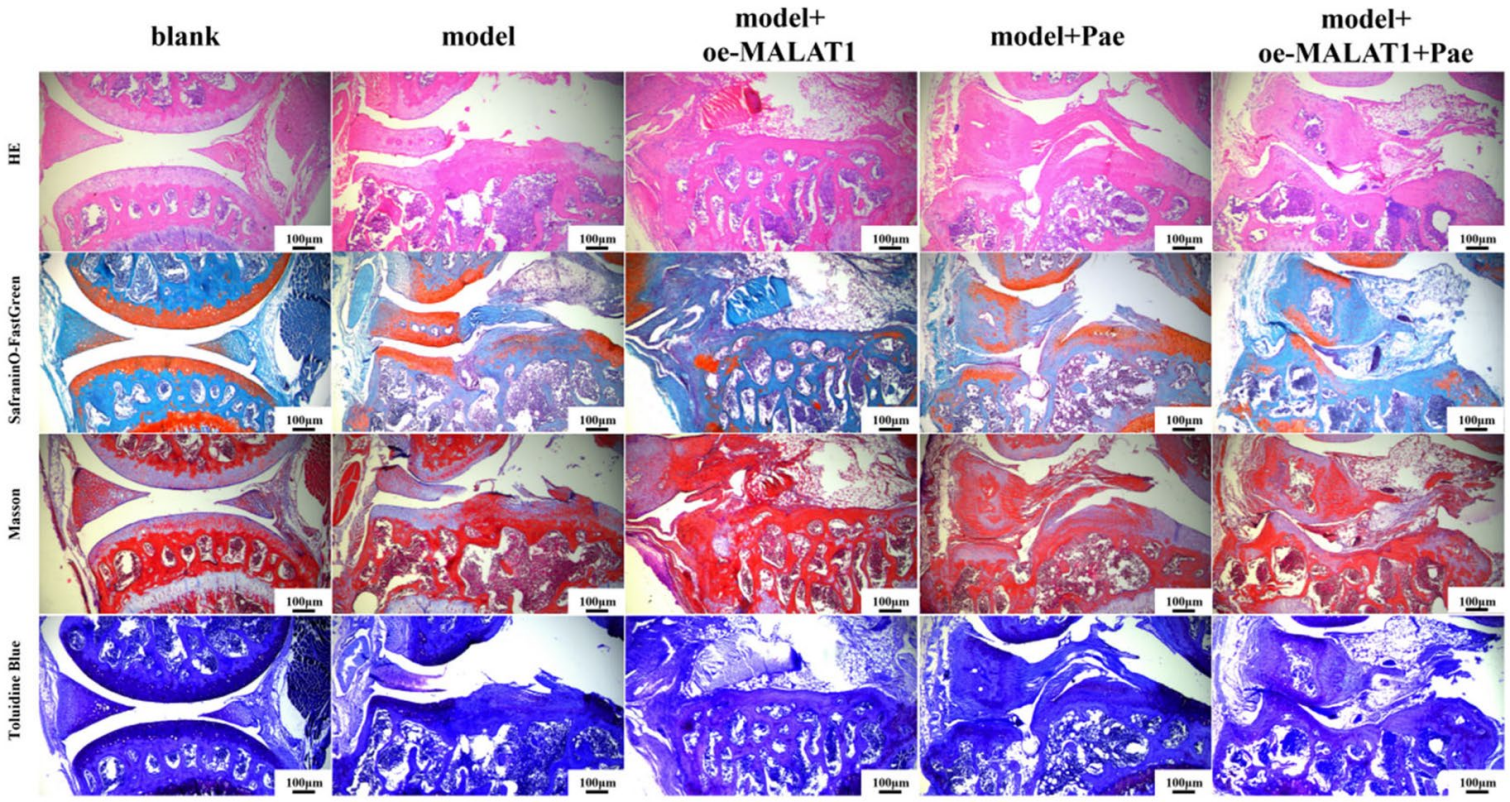
A-D: Histomorphological alterations in the knee joint *via* HE (a), SafraninO-FastGreen (B), Toluidine Blue (C), and masson (D) staining. Magnification: 100×.

We applied HE, SafraninO-FastGreen, Toluidine Blue, and Masson’s trichrome staining to evaluate morphological changes in the articular cartilage of OA mice and to confirm the protective effects of Pae. In the blank group, cartilage layers appeared smooth and continuous, with an intact extracellular matrix and normal chondrocyte morphology. In contrast, the model group exhibited marked structural disruption, including discontinuous cartilage layers, severe matrix degradation, and exposed chondrocytes. The model + Pae group showed preservation of overall cartilage architecture, with largely intact cartilage layers. Compared with the model + oe-MALAT1 group, the model + oe-MALAT1 + Pae group displayed improved structural integrity of the cartilage layer, indicating that Pae mitigated cartilage damage even under MALAT1 overexpression (Figure 1).

### Pae regulates lncRNA MALAT1 expression levels and cholesterol-oxidative stress-iron-eelated genes in cartilage tissue

**Figure 2.**
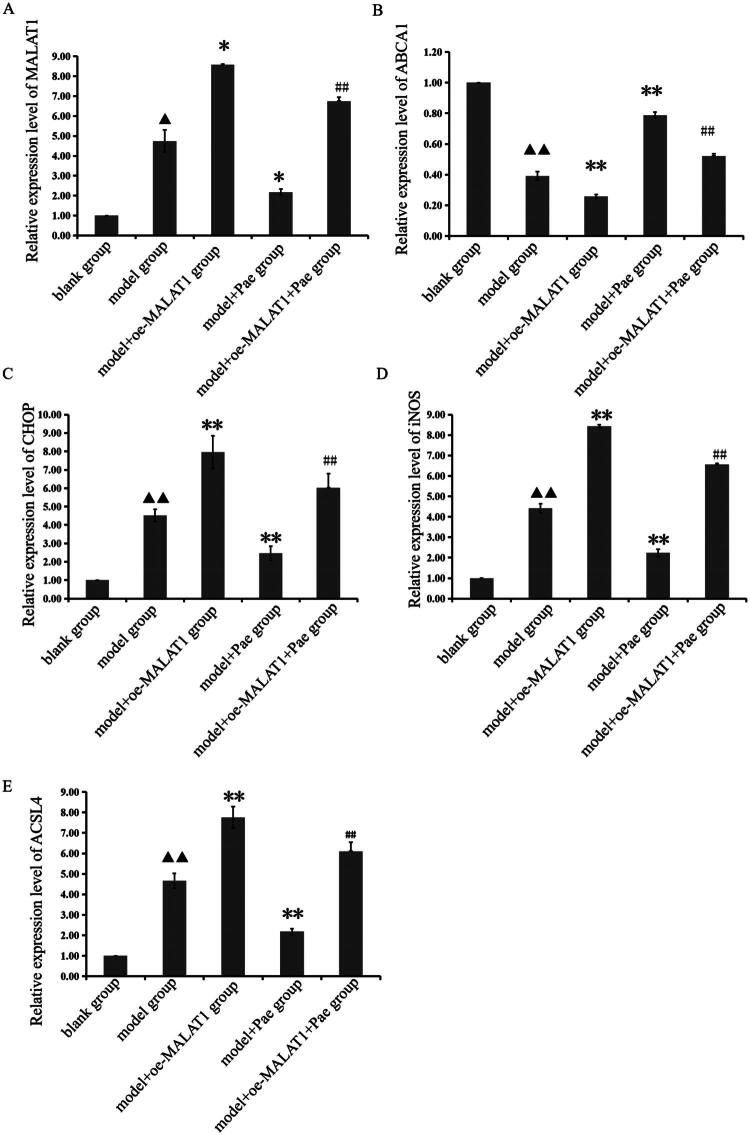
Relative expression levels of lncRNA MALAT1 and related genes in cartilage tissues across groups. Overexpression of the lncRNA MALAT1 modulates effects of pae. ^▲▲^*p* < 0.01 or ^▲^*p* < 0.05 compared with the blank group; **p < 0.01 or *p < 0.05 compared with the model group; ^##^*p* < 0.01 compared with the model + pae group. Data are representative of three independent biological experiments.

To determine whether Pae influences the role of lncRNA MALAT1 in OA through cholesterol-oxidative stress-iron metabolic dysregulation, we overexpressed lncRNA MALAT1 and quantified the expression of lncRNA MALAT1, ABCA1, CHOP, iNOS, and ACSL4 in mouse cartilage tissue using real-time PCR. Relative to the blank group, the model group displayed significantly elevated expression of lncRNA MALAT1, CHOP, iNOS, and ACSL4, whereas ABCA1 expression was markedly reduced (*p* < 0.05). Compared with the model group, Pae treatment resulted in a substantial reduction in lncRNA MALAT1, CHOP, iNOS, COX2, and ACSL4 expression, while ABCA1 expression was significantly upregulated (*p* < 0.05). Furthermore, compared with the model+Pae group, the model+oe-MALAT1+Pae group exhibited increased expression of lncRNA MALAT1, CHOP, iNOS, and ACSL4, along with decreased ABCA1 expression (*p* < 0.01, Figure 2). These findings demonstrate that lncRNA MALAT1 is highly expressed in OA cartilage and contributes to cholesterol-oxidative stress-iron metabolic imbalance. Pae reverses these pathological changes; however, its beneficial effects are partially suppressed by lncRNA MALAT1 overexpression.

### Pae improves expression of related proteins in OA cartilage tissue

**Figure 3.**
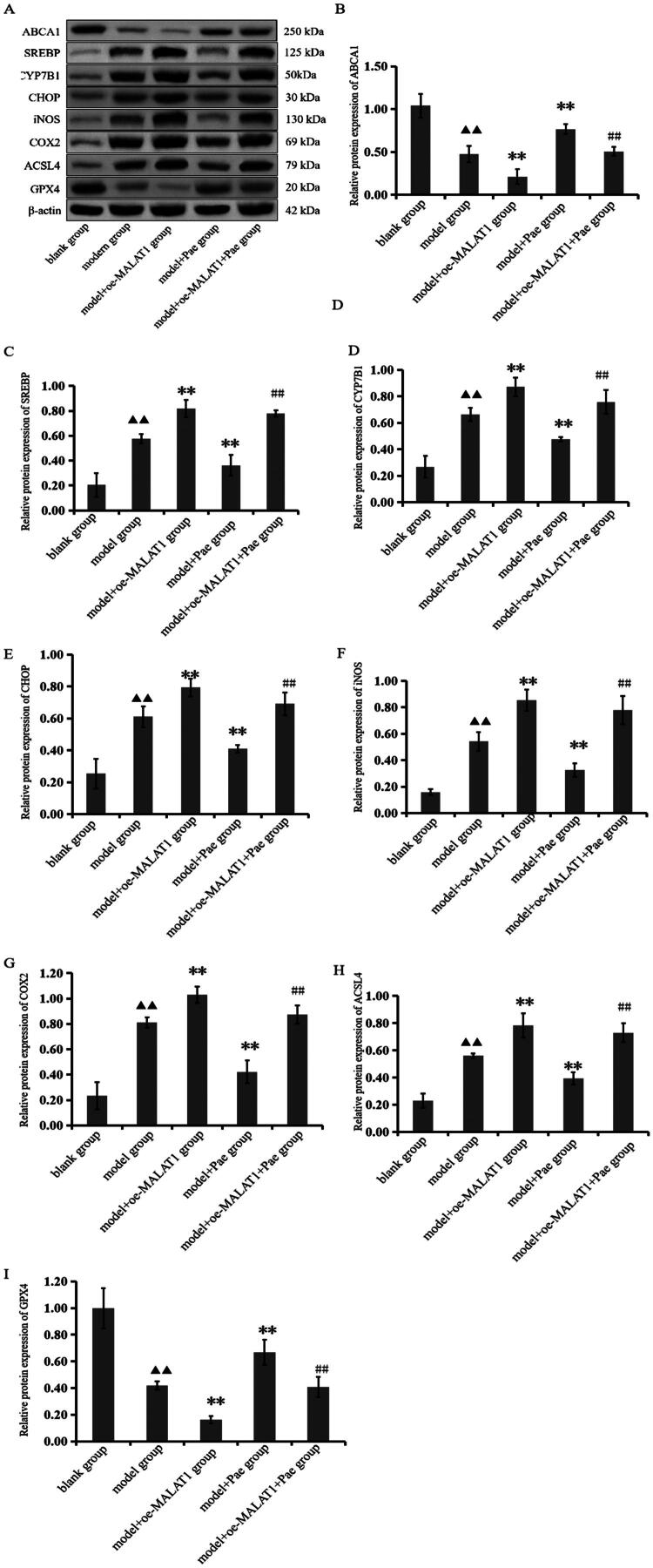
Protein expression of ABCA1, SREBP, CYP7B1, CHOP, iNOS, COX2, ACSL4, and GPX4 in cartilage tissue across groups following pae intervention. A: Electrophoretic patterns of ABCA1, SREBP, CYP7B1, CHOP, iNOS, COX2, ACSL4, and GPX4 proteins. B-I: Quantitative analysis of protein band intensities for ABCA1, SREBP, CYP7B1, CHOP, iNOS, COX2, ACSL4, and GPX4. ^▲▲^*p* < 0.01 compared with the blank group; **p < 0.01 compared with the model group; ^##^*p* < 0.01 compared with the model + pae group. Data are representative of three independent biological experiments.

We performed western blot analysis to examine the expression of cholesterol-oxidative stress-iron metabolic-related proteins, including ABCA1, SREBP, CYP7B1, CHOP, iNOS, COX2, ACSL4, and GPX4, in cartilage tissue from OA mice. Compared with the blank group, the model group exhibited a marked reduction in ABCA1 and GPX4 expression, along with significant increases in SREBP, CYP7B1, CHOP, iNOS, COX2, and ACSL4 protein levels (*p* < 0.05). Treatment with Pae reversed these changes: relative to the model group, the model + Pae group showed substantial upregulation of ABCA1 and GPX4 and a pronounced reduction in SREBP, CYP7B1, CHOP, iNOS, COX2, and ACSL4 expression (*p* < 0.01). Furthermore, compared with the model+Pae group, the model+oe-MALAT1+Pae group exhibited significantly decreased levels of ABCA1 and GPX4, whereas the expression of SREBP, CYP7B1, CHOP, iNOS, COX2, and ACSL4 was markedly increased (*p* < 0.01, Figure 3). These results indicate that Pae modulates the expression of cholesterol-oxidative stress-iron-related proteins in OA cartilage tissue, and this regulatory effect is closely associated with lncRNA MALAT1.

### Pae alleviates total iron accumulation in OA cartilage tissue

**Figure 4.**
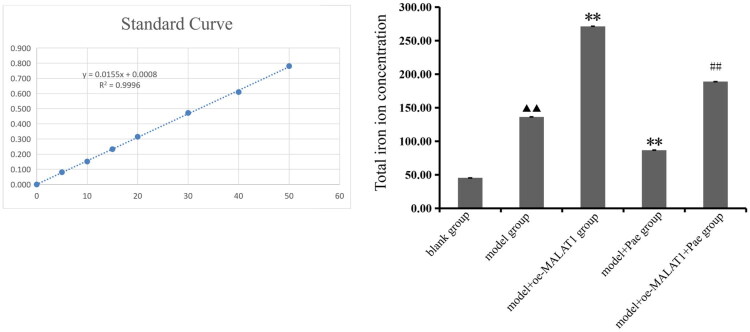
Total iron levels in each group after pae treatment. ^▲▲^*p* < 0.01 compared with the blank group; **p < 0.01 compared with the model group; ^##^*p* < 0.01 compared with the model + pae group. Data are representative of three independent biological experiments.

The total iron levels in mouse bone tissues were determined using a total iron assay kit. The results showed that, compared with the control group, total iron levels were significantly increased in the model group (*p* < 0.01). In contrast, total iron levels were markedly reduced in the model + Pae group compared with the model group (*p* < 0.01). Furthermore, compared with the model + Pae group, total iron levels were significantly elevated in the model + oe-MALAT1 + Pae group (*p* < 0.01; Figure 4).

### Pae inhibits IL-1β-induced expression of lncRNA MALAT1 in chondrocytes

**Figure 5.**
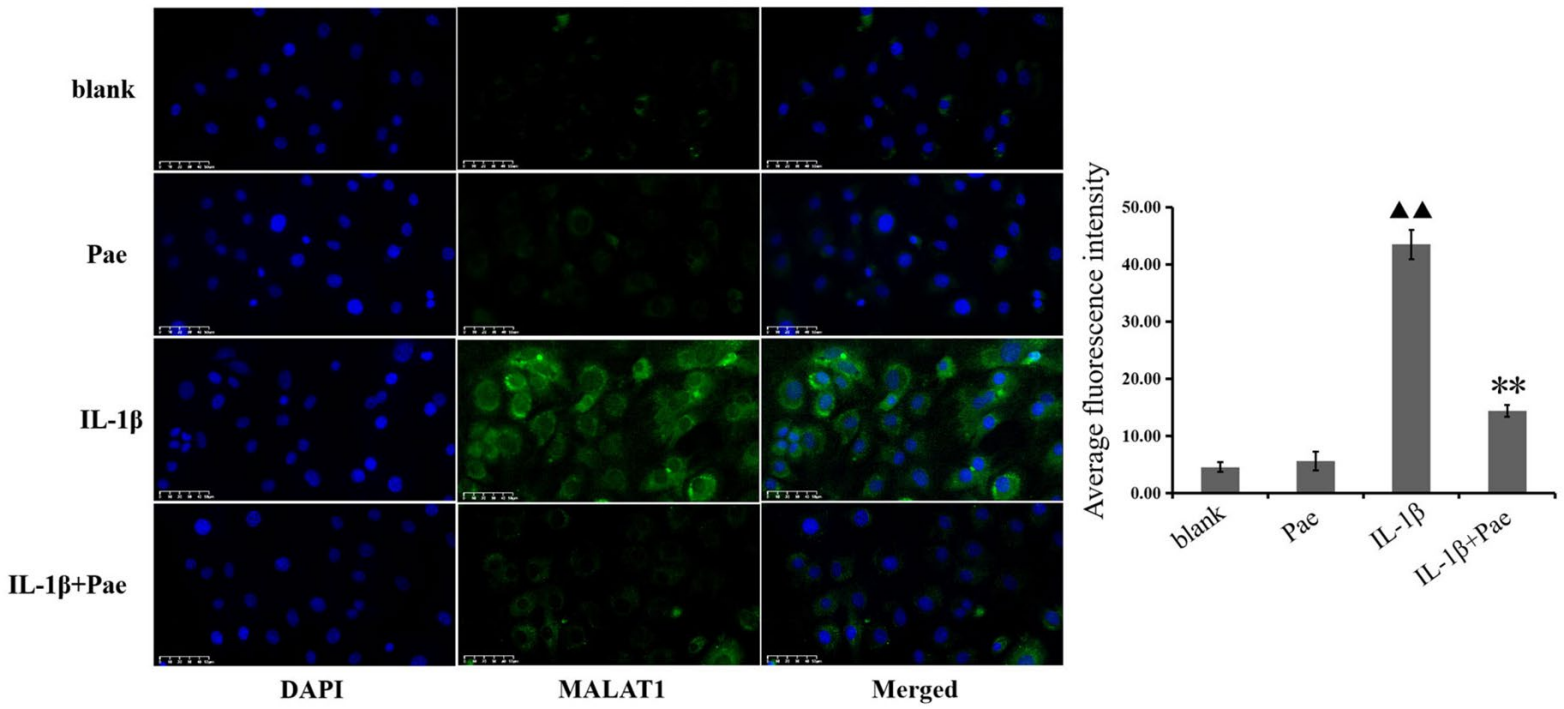
Pae inhibits IL-1β-induced lncRNA MALAT1 expression in chondrocytes. A: Fluorescence intensities of lncRNA MALAT1 in chondrocytes across groups (400×). B: Fluorescence analysis of lncRNA MALAT1 in chondrocytes across groups. ^▲▲^*p* < 0.01 compared with the blank group; **p < 0.01 compared with the IL-1β group. Data are representative of three independent biological experiments.

To further confirm the involvement of lncRNA MALAT1 in the Pae-mediated regulation of the OA cholesterol-oxidative stress-iron metabolic pathway, we performed *in vitro* experiments using well-cultured F0 chondrocytes. FISH analysis demonstrated that lncRNA MALAT1 copy numbers were markedly elevated in chondrocytes after IL-1β stimulation (*p* < 0.01), as evidenced by strong green fluorescence signals. Pae treatment significantly reduced lncRNA MALAT1 expression, with a notable decline in average fluorescence intensity (*p* < 0.01, Figure 5).

### Knockdown of lncRNA MALAT1 affects expression of lncRNA MALAT1, ABCA1, CHOP, iNOS, and ACSL4 in pae-treated IL-1β-induced chondrocytes

**Figure 6.**
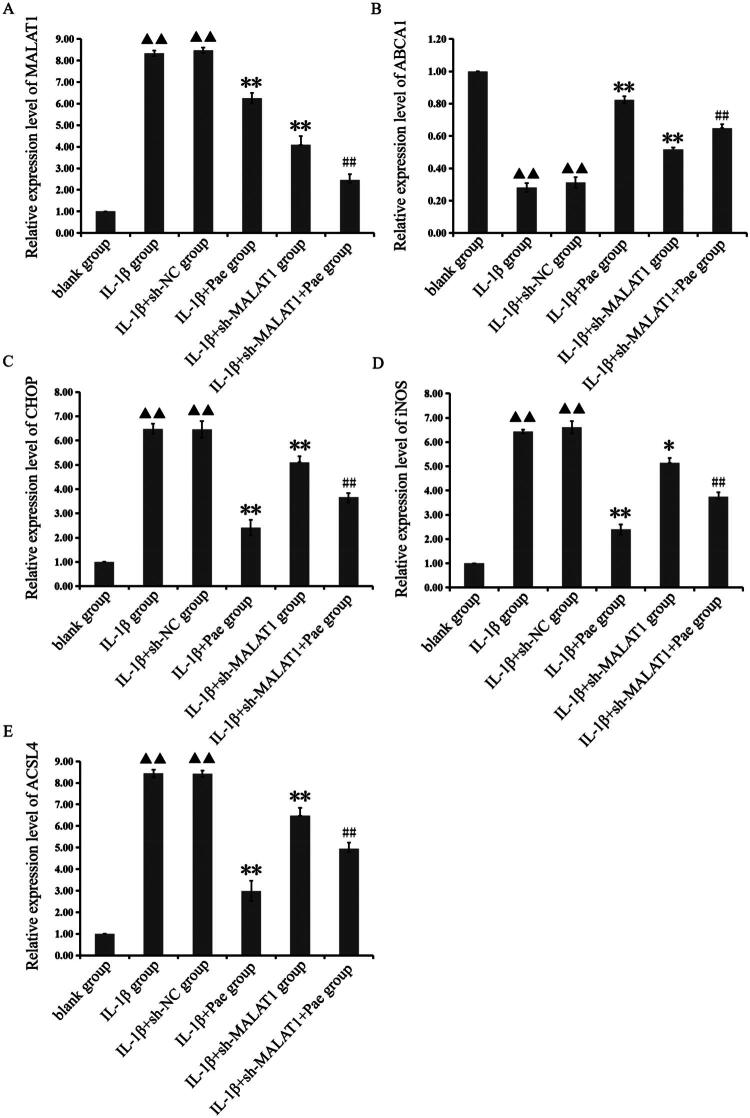
Changes to lncRNA MALAT1 expression levels following sh-MALAT1 transfection in IL-1β-induced chondrocytes. ^▲▲^*p* < 0.01 compared with the blank group; **p < 0.01 or *p < 0.05 compared with the IL-1β group; ^##^*p* < 0.01 compared with the IL-1β + sh-MALAT1 group. Data are representative of three independent biological experiments.

Real-time PCR analysis revealed that IL-1β stimulation significantly altered gene expression in chondrocytes. Compared with the blank group, IL-1β-treated chondrocytes displayed markedly elevated expression of lncRNA MALAT1, iNOS, CHOP, and ACSL4, while ABCA1 expression was significantly reduced (*p* < 0.01). Following sh-MALAT1 transfection, lncRNA MALAT1 levels decreased in IL-1β + sh-MALAT1 chondrocytes compared with the IL-1β group (*p* < 0.01), confirming successful knockdown. However, CHOP, iNOS, and ACSL4 expression remained significantly elevated, while ABCA1 expression was further reduced (*p* < 0.05). Importantly, compared with the IL-1β + sh-MALAT1 group, the IL-1β + sh-MALAT1 + Pae group exhibited suppressed expression of lncRNA MALAT1, CHOP, iNOS, and ACSL4, whereas ABCA1 expression was significantly upregulated (*p* < 0.01, Figure 6). These findings indicate that Pae regulates lncRNA MALAT1, ABCA1, CHOP, iNOS, and ACSL4 expression in IL-1β-induced mouse chondrocytes, suggesting a functional interaction between lncRNA MALAT1 expression and Pae activity.

### Pae mediates lncRNA MALAT1 to improve OA cholesterol-oxidative stress-iron metabolic dysregulation

**Figure 7.**
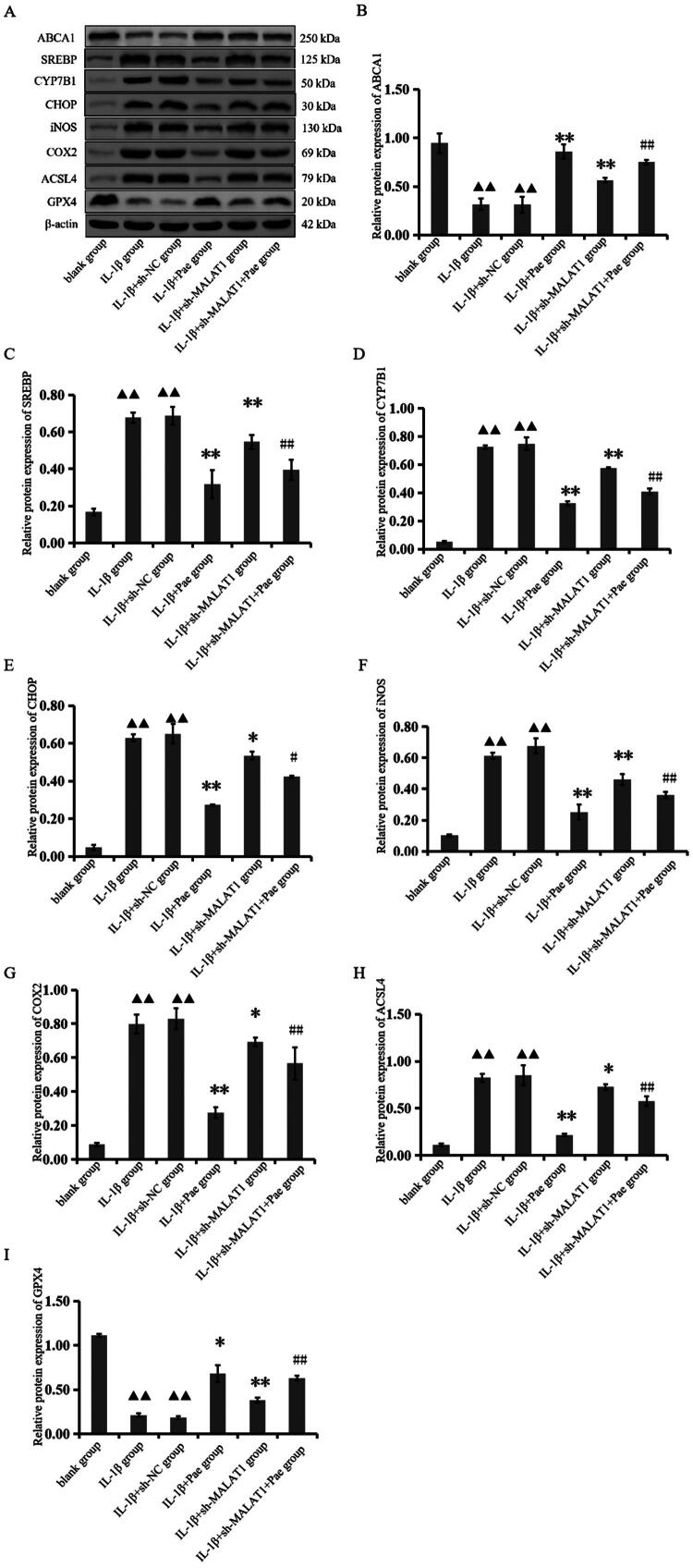
Expression of lncRNA MALAT1 modulates pae’s effects on cholesterol-oxidative stress-iron metabolism-related proteins in chondrocytes. ^▲▲^*p* < 0.01 compared with the blank group; **p < 0.01 or *p < 0.05 compared with the IL-1β group; ^##^*p* < 0.01 or ^#^*p* < 0.05compared with the IL-1β + sh-MALAT1 group. Data are representative of three independent biological experiments.

To further validate that Pae ameliorates OA chondrocyte cholesterol-oxidative stress-iron metabolic dysfunction *via* lncRNA MALAT1, we performed western blot analysis under lncRNA MALAT1 knockdown conditions. Compared with the IL-1β group, the IL-1β + Pae group exhibited significantly increased ABCA1 and GPX4 levels, whereas SREBP, CYP7B1, CHOP, iNOS, COX2, and ACSL4 expression was markedly reduced (*p* < 0.05). Moreover, relative to the IL-1β + sh-MALAT1 group, the IL-1β + sh-MALAT1 + Pae group showed pronounced downregulation of SREBP, CYP7B1, CHOP, iNOS, COX2, and ACSL4, along with significant upregulation of ABCA1 and GPX4 (*p* < 0.05, Figure 7). Together, these results demonstrate that MALAT1 actively participates in the OA cholesterol-oxidative stress-iron metabolic pathway, and Pae exerts protective effects by modulating this lncRNA MALAT1-dependent mechanism.

### Pae inhibits IL-1β-induced chondrocyte apoptosis rates

**Figure 8.**
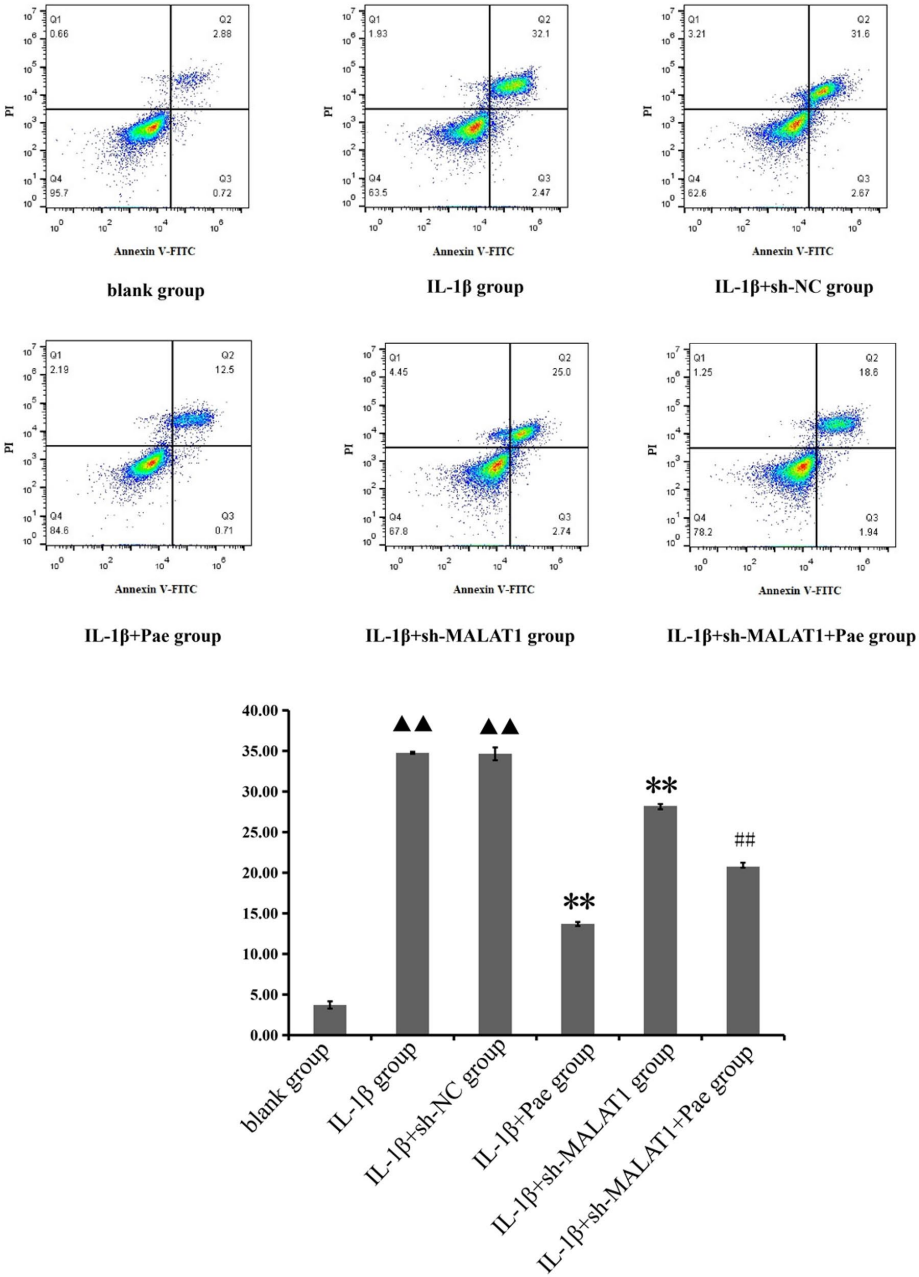
Apoptosis in chondrocytes across groups and associated analyses. ^▲▲^*p* < 0.01 compared with the blank group; **p < 0.01 compared with the IL-1β group; ^##^*p* < 0.01 compared with the IL-1β+ sh-MALAT1 group. Data are representative of three independent biological experiments.

Flow cytometry analysis showed that IL-1β treatment significantly increased chondrocyte apoptosis (*p* < 0.01). Pae intervention markedly reduced apoptosis, and this protective effect was further enhanced when lncRNA MALAT1 was silenced. These findings indicate that Pae decreases chondrocyte apoptosis by regulating lncRNA MALAT1 expression (Figure 8). This demonstrates that Pae can reduce chondrocyte apoptosis by regulating the expression level of lncRNA MALAT1.

## Discussion

LncRNA MALAT1 plays a pivotal regulatory role in subchondral bone, synovium, cartilage tissue, and chondrocytes in OA, closely associated with chondrocyte apoptosis, proliferation, extracellular matrix (ECM) synthesis and degradation, and inflammatory responses (Wang et al., 2022). Notably, lncRNA MALAT1 expression is upregulated in obesity-associated OA, highlighting its involvement in metabolic dysregulation (Nanus et al., 2020). Our previous work demonstrated that aberrant lncRNA MALAT1 expression disrupts cholesterol homeostasis and iron metabolism, thereby influencing OA progression (Fu et al., 2025). Furthermore, recent evidence indicates that cholesterol metabolism can directly induce intracellular oxidative stress, which serves as a key upstream driver of ferroptosis, suggesting a tightly connected metabolic axis involving cholesterol, oxidative stress, and iron dyshomeostasis (Wu et al., 2025; Zhang et al., 2022).

Ferroptosis, an iron-dependent form of non-apoptotic cell death, is primarily triggered by lipid peroxidation (Dixon et al., 2012; Chen et al., 2021). GPX4, a central regulator of ferroptosis, is downregulated in OA chondrocytes, leading to iron accumulation, ECM degradation, and osteophyte formation (Miao et al., 2022). In OA mouse models, iron overload disrupts iron homeostasis, increases intracellular ROS and lipid ROS, and downregulates ACSL4, ultimately preserving ECM and delaying OA progression (Yao et al., 2021). Cholesterol transport by ABCA1 and regulation by SREBP2 are critical for maintaining chondrocyte lipid homeostasis; ABCA1 upregulation prevents cholesterol accumulation, reduces ECM degradation, and inhibits apoptosis (Fu et al., 2024), while modulation of SREBP2 expression mitigates lipid metabolic dysregulation, oxidative stress, and inflammation in KOA rats (Gundogdu et al., 2024). In addition, CYP7B1 knockout can correct cholesterol metabolic disturbances in chondrocytes, reducing catabolic activity and improving OA pathology (Choi et al., 2019). CHOP silencing has been reported to activate ferroptotic pathways, promoting intracellular iron accumulation and lipid peroxidation (Wang et al., 2025), whereas inhibition of iNOS and COX2 decreases ROS generation, alleviates oxidative stress, and improves synovitis, cartilage degeneration, and bone remodeling in OA (Lv et al., 2025).

Recent studies have demonstrated that Pae exerts protective effects in OA through its antioxidant, anti-inflammatory, and anti-apoptotic activities (Jiao et al., 2021; Liao et al., 2018). Pae may indirectly modulate lncRNA MALAT1 expression *via* downregulation of transcription factors. Our dual-luciferase assays revealed that miR-16-5p directly targets lncRNA MALAT1, forming a competitive endogenous RNA (ceRNA) network that regulates downstream genes, including ACSL4 (Fu et al., 2025; Fang et al., 2023). Network pharmacology and molecular docking further indicated that Pae could interact with ABCA1, SREBP, CHOP, iNOS, COX2, and GPX4, thereby influencing cholesterol metabolism, oxidative stress, and iron homeostasis. Importantly, Pae can alleviate iron overload-induced chondrocyte injury, inhibit ferroptosis, and mitigate iron-related OA progression (Wu et al., 2025). However, whether Pae directly regulates the “cholesterol-oxidative stress-iron” metabolic axis in OA remains unclear.

Morphological staining in this *in vivo* investigation revealed that mice treated with Pae exhibited less structural damage to joint cartilage when lncRNA MALAT1 was overexpressed. However, real-time PCR results showed that Pae intervention significantly downregulated lncRNA MALAT1 expression, indicating that Pae may influence the pathophysiology of OA through regulation of lncRNA MALAT1. Western blot research has consistently shown that whereas lncRNA MALAT1 overexpression reduced Pae’s protective effects, Pae increased the expression of proteins associated with the cholesterol-oxidative stress-iron axis in cartilage tissue. These findings suggest that Pae alleviates OA progression, at least in part, by modulating lncRNA MALAT1 and its downstream pathways. Total iron measurements revealed that Pae intervention alleviates iron accumulation in bone tissue and significantly reduces total iron levels, indicating that Pae can regulate iron homeostasis and mitigate iron overload. To investigate whether Pae regulates lncRNA MALAT1 to improve lipid metabolism, inhibit ferroptosis, and reduce oxidative stress in osteoarthritic chondrocytes, we established an *in vitro* chondrocyte model treated with IL-1β and co-cultured it with lncRNA MALAT1 knockdown mouse chondrocytes. FISH analysis showed that IL-1β significantly increased lncRNA MALAT1 fluorescence intensity in chondrocytes, indicating elevated expression, whereas Pae treatment reversed this effect and markedly reduced fluorescence intensity. Real-time PCR further confirmed these findings: IL-1β stimulation significantly upregulated lncRNA MALAT1 expression in chondrocytes, while Pae intervention effectively suppressed it. Western blot analysis revealed that Pae not only improved lipid metabolism but also regulated ferroptosis and oxidative stress pathways. Pae exerted regulatory effects on lipid metabolism regulators ABCA1, SREBP2, and CYP7B1, ferroptosis-related proteins GPX4 and ACSL4, and oxidative stress-related proteins CHOP, iNOS, and COX2. Notably, lncRNA MALAT1 knockdown altered the magnitude of Pae’s protective effects, underscoring MALAT1’s key role in mediating Pae’s chondroprotective function. These findings elucidate the molecular mechanism by which Pae exerts therapeutic benefits in OA. lncRNA MALAT1 exacerbates metabolic dysfunction in chondrocytes by driving disturbances in cholesterol homeostasis, oxidative stress, and iron overload. Both *in vivo* and *in vitro*, we found that MALAT1 regulates the expression of ABCA1, SREBP, CYP7B1, ACSL4, GPX4, CHOP, iNOS, and COX2 in cartilage tissue and chondrocytes. Pae intervention reverses these pathological changes, restores lipid metabolism, suppresses ferroptosis, and reduces oxidative stress, thereby protecting joint cartilage integrity and delaying OA progression.

## Conclusions

Taken together, this study not only elucidates the molecular basis by which Paeoniflorin mitigates osteoarthritic cartilage degeneration but also highlights its potential translational relevance. Our findings indicate that Paeoniflorin alleviates lncRNA MALAT1-associated cholesterol-oxidative stress-iron metabolic dysregulation in osteoarthritic chondrocytes.

Nevertheless, several limitations should be acknowledged. Although the present findings were consistently validated using established *in vivo* and *in vitro* OA models, the lack of confirmation in human chondrocytes or clinical cartilage specimens limits direct extrapolation to human disease. Future studies incorporating human-derived chondrocytes and clinical samples will be essential to further substantiate the translational potential of targeting lncRNA MALAT1-centered metabolic imbalance in osteoarthritis.

## Supplementary Material

Supplemental Material

## Data Availability

The data that support the findings of this study are available from the corresponding author, Lin, upon reasonable request.
