# Supplementary figures and images for: lncRNA MALAT1-mediated regulation of cholesterol-oxidative stress-iron metabolic dysregulation by paeoniflorin in osteoarthritic chondrocytes
Source: Pharm Biol. 2026 Feb 12;64(1):278–94. doi: 10.1080/13880209.2026.2620862 (PMC12912232; doi:10.1080/13880209.2026.2620862)

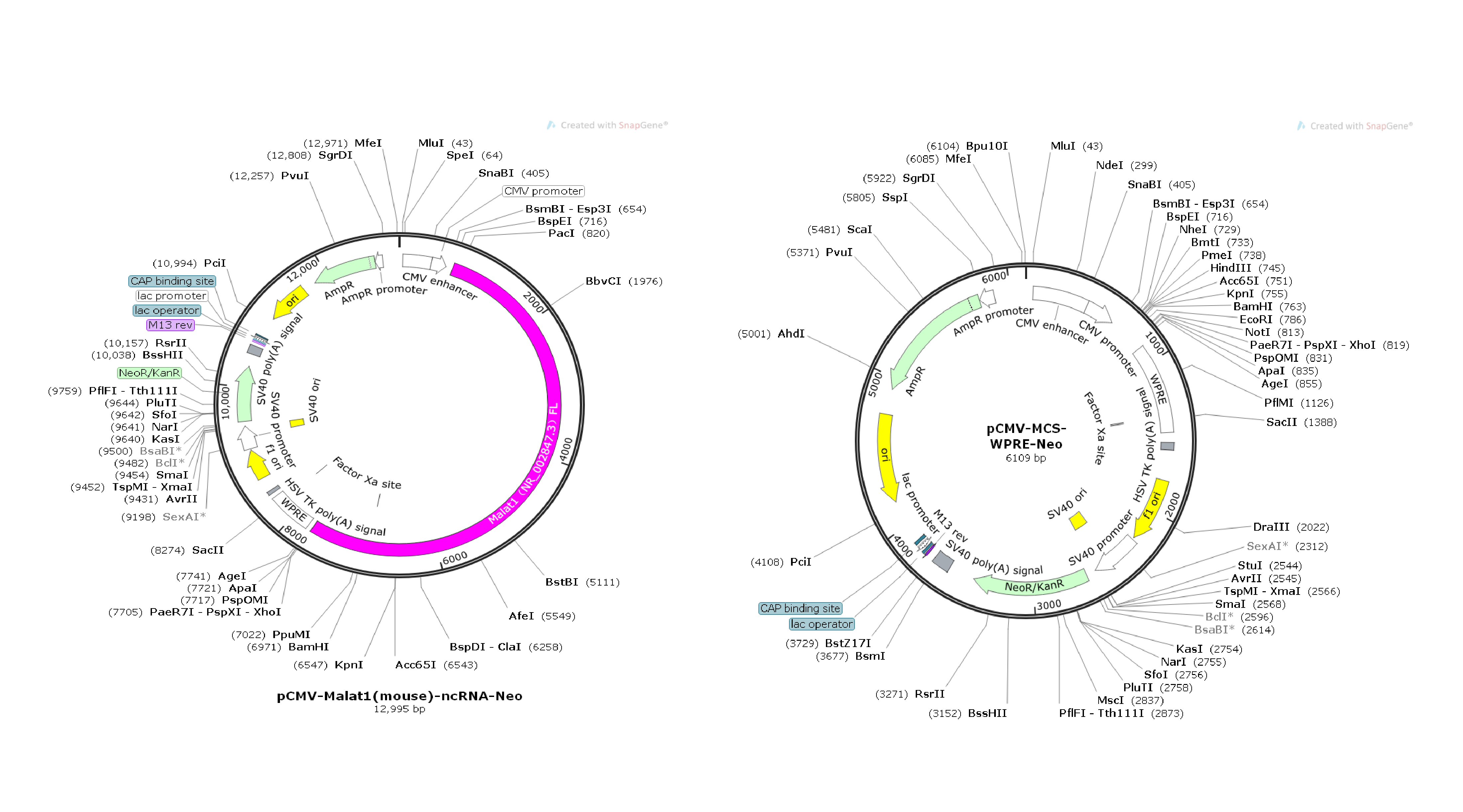
Figure S1. Plasmid map

Supplement: Supplemental Material [file IPHB_A_2620862_SM9102.docx]
